# Supplementary material for: Impact of ATM and DNA-PK Inhibition on Gene Expression and Individual Response of Human Lymphocytes to Mixed Beams of Alpha Particles and X-Rays
Source: Cancers (Basel). 2019 Dec 13;11(12):2013. doi: 10.3390/cancers11122013 (PMC6966634; doi:10.3390/cancers11122013)
Supplement: Supplementary file 1 [file cancers-11-02013-s001.zip › cancers-647312-Figure S2.pdf]

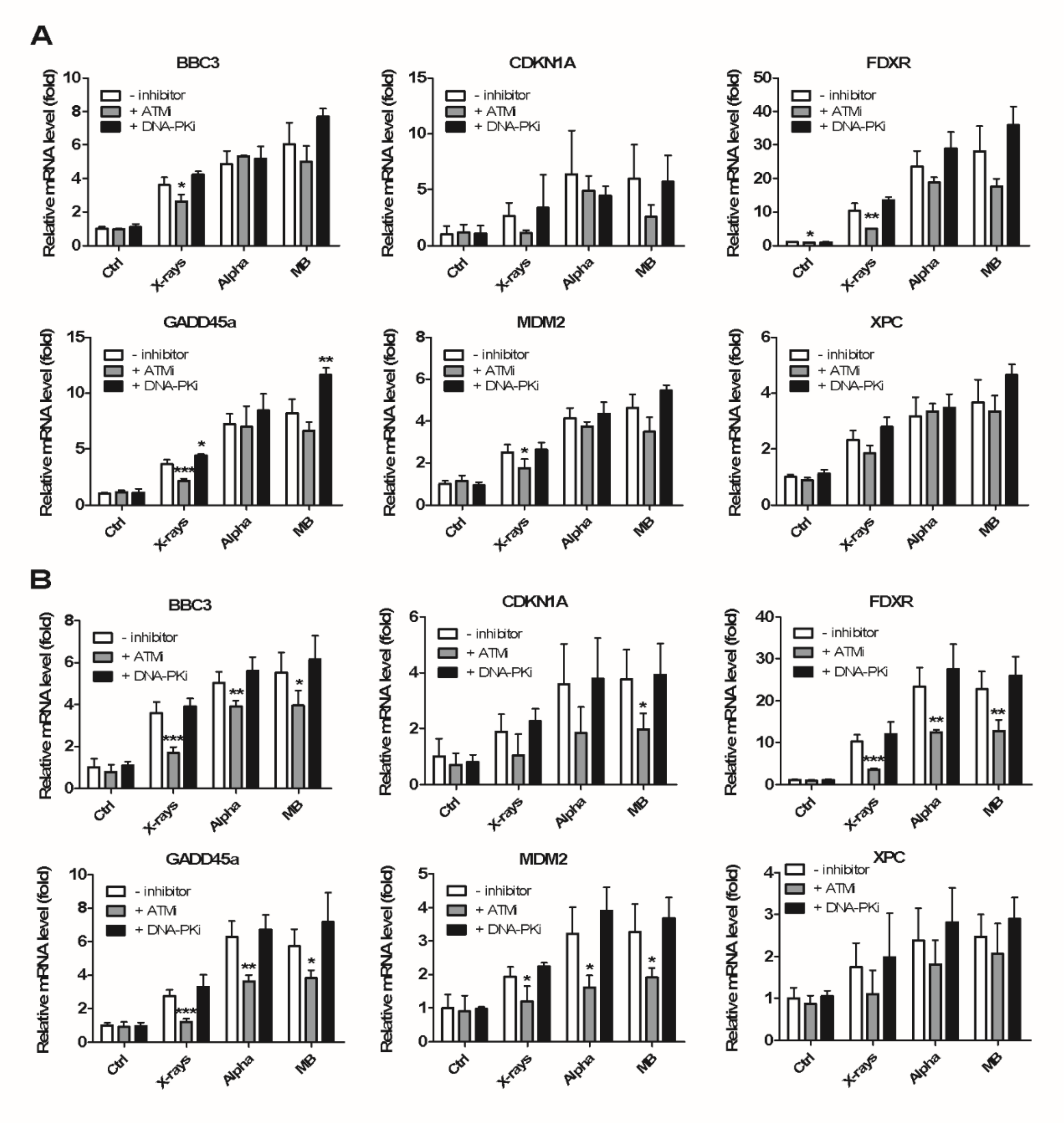

Supplemental figure 2. Gene expression levels in PBL 24 h after exposure to 1 Gy X-rays, alpha particles or mixed beams, respectively, with or without inhibition of ATM (KU55933) or DNA-PK (NU7026) in Donor 3 (**a**) and 4 (**b**). Mean results from 3 experiments are presented, relative to average control without inhibitor. Error bars represent standard deviations. Asterisks represent significant differences at the level of *< 0.05, **<0.01 and ***<0.001 of mRNA levels in PBL 24 h after the same exposure with inhibitor compared to without inhibitor (student t-test).
